# Oral Manifestations in Children Diagnosed with COVID-19: A Narrative Review

**DOI:** 10.3390/healthcare11030288

**Published:** 2023-01-17

**Authors:** Abel Emanuel Moca, Raluca Iulia Juncar, Rahela Tabita Moca, Teofana Bota, Denisa Tabita Sabău, Mihai Juncar

**Affiliations:** 1 Department of Dentistry, Faculty of Medicine and Pharmacy, University of Oradea, 10 Piața 1 Decembrie Street, 410073 Oradea, Romania; 2 Doctoral School of Biomedical Sciences, University of Oradea, 1 Universității Street, 410087 Oradea, Romania

**Keywords:** COVID-19, oral manifestations, children, adolescents, pandemic

## Abstract

The COVID-19 disease has many symptoms, including fever, dry cough, tachypnea, and shortness of breath, but other symptoms can accompany the disease. The disease can also have oral manifestations. The aim of this narrative review is to describe the oral manifestations of COVID-19 in children and adolescents by summarizing the current knowledge as it was described in various case reports and original articles. A review of the literature was carried out by searching the online databases PubMed, Web of Science and Scopus, between October 2022 and 12 November 2022. For this narrative review, 890 articles from three databases and manual search were screened. Saliva was discovered to be a potential screening tool for the infection with the SARS-CoV-2, although it is most reliable in the first few days of infection. Different alteration of the oral mucosa, such as ulcers, erosions and gingivitis were reported. Oral manifestations accompanied children with COVID-19-related multisystem inflammatory syndrome, Kawasaki disease, thrombocytopenic purpura and erythema multiforme. COVID-19 had an indirect effect on oral harmful habits by decreasing their frequency during the lockdown. Although they occur more rarely, oral manifestations can accompany COVID-19 disease in children and adolescents, and they can be an early sign of the disease.

## 1. Introduction

The virus that causes the COVID-19 disease has a high transmission rate that occurs through respiratory droplets, saliva, coughing, sneezing, breathing and speech, both from symptomatic and asymptomatic patients [1,2]. Due to the increase in the number of deaths and new infections, the World Health Organization (WHO) declared a global pandemic of COVID-19 and suggested a series of restrictive measures in order to reduce the spread of the virus [3].

Among the first symptoms of the disease are fever, dry cough, tachypnea, and shortness of breath [4], but the clinical picture of the disease can be supplemented with various symptoms, such as chest pain, vomiting, diarrhea, nasal congestion, sputum production, anosmia, dyspepsia, viral conjunctivitis, or even sepsis [5,6]. In children, the symptoms are generally less severe than in adults [7]. After the incubation period, children may not develop any symptoms, and if they appear, they are generally represented by fever and cough, but they may also develop digestive or nervous system symptoms [7,8]. However, there are rare situations in which children and adolescents can develop severe clinical forms, and the disease can have a fatal outcome [9].

COVID-19 can have an impact on the oral cavity as well, with dysgeusia being the most well-known oral symptom [10], but the disease can also have other visible oral manifestations. In adults, the development of ulcers, erosions, vesicles, pustules, halitosis, pigmentation and numerous other oral manifestations have been described [11]. Oral manifestations of COVID-19 should be identified, giving the fact that a large number of general diseases show oral mucosal alterations [12], and that macule, papule, erosions, or ulcers have been described in viral, bacterial, fungal, and parasitic diseases affecting children [13]. Moreover, most viral infections tend to have oral manifestations, which can present as an early sign of the disease [14].

Even though new data on COVID-19 are constantly increasing both in terms of treatment, but also in the clinical manifestations of the disease [15], up until the moment of designing this study, no complex review articles have been identified that address the oral manifestations of COVID-19 in children, in general. Acquiring new information on the clinical picture of COVID-19 is necessary and of crucial importance in the long term.

The aim of this narrative review is to describe the oral manifestations of COVID-19 in children and adolescents by summarizing the current knowledge as it was described in various case reports and original articles.

## 2. Materials and Methods

### 2.1. Search Strategy

A review of the literature was carried out regarding the oral manifestations identified in children and adolescents with COVID-19. The search was conducted on the online databases PubMed, Web of Science and Scopus, using the following keywords: “oral manifestations” or “oral lesions” or “oral diseases” and “COVID-19” or “SARS-CoV-2” and “children” or “pediatric”. Articles published between January 2020 and September 2022 were screened. The search was conducted between 20 October 2022 and 12 November 2022.

### 2.2. Study Selection and Eligibility Criteria

The authors carefully checked the articles and selected the most relevant ones for this review. All articles were checked independently by two authors. Articles containing the aforementioned keywords were included in this manuscript. The inclusion criteria were represented by retrospective studies that identified the oral manifestations of COVID-19 in children and adolescents, case reports of some child or adolescent patients diagnosed with COVID-19 who had manifestations in the oral cavity, and letters to the editor and brief communications that identified oral manifestations in children and adolescents diagnosed with COVID-19. Systematic reviews, meta-analyses or other types of reviews, as well as animal studies were excluded. Studies that did not meet the requirements were excluded.

**Figure 1:**
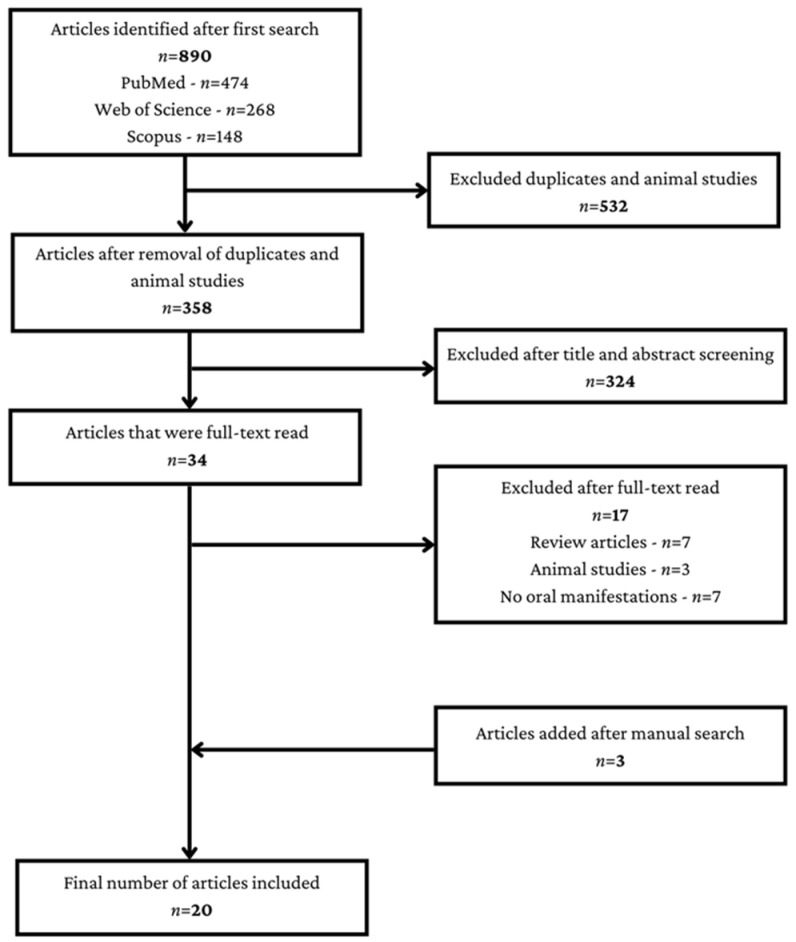
Study selection process.

The Initial search retrieved 890 articles from PubMed, Web of Science and Scopus, of which 532 were duplicates or animal studies and were immediately excluded. Out of the 358 remaining articles, an additional 324 were excluded, and after full-text read, another 17 articles were excluded. To the remaining 17 articles, an additional 3 articles were added after manual search. Finally, 20 articles were included in this review (Figure 1).

## 3. Narrative Synthesis

### 3.1. Saliva as a Diagnostic Tool

The diagnosis of SARC-CoV-2 infection applies reverse transcriptase polymerase chain reaction (RT-PCR) to the samples obtained by nasopharyngeal and oropharyngeal swab, this method having an excellent diagnostic accuracy [16]. However, the costs for this diagnostic method are high and time-consuming, so that faster and cheaper diagnostic methods have been researched [17].

Saliva is a hypotonic solution that contains 99% water, and the remaining 1% is represented by various organic and inorganic substances [18]. The diagnosis of various general pathologies through saliva is considered very promising, because it would allow an early diagnosis as well as an excellent monitoring of biomarkers [19]. Viral infections can be diagnosed from saliva either directly, by targeting the genetic material of the virus, or indirectly, by proteomic analysis, metabolic analysis, or immunoglobulin profiles [20]. Saliva is easy to collect, and it can be a reservoir of biomarkers for the detection of SARS-CoV-2 infection [21], considering the fact that the virus has been consistently identified in saliva [22].

A study that investigated the possibility of reporting the presence of the SARS-CoV-2 virus in the saliva of children and adolescents was identified. The authors collected saliva samples from four pediatric patients (two children and two adolescents) but also from an adult patient diagnosed positive for the SARS-CoV-2 virus. Each adolescent patient provided one saliva sample, but children provided more saliva samples. For viral identification from saliva, the authors performed viral RNA extraction with spin column-based Kit RNAv-090 from BioPure. The authors found that the initial diagnostic test from the nasopharynx collection (NP) had a 100% agreement with the one from the saliva obtained in the first sample, and the authors could easily identify the viral genome in the saliva. One of the samples obtained on the third day after diagnosis was negative, which may suggest that the test is not 100% safe even in the first days of infection [23].

Han et al. (2020) collected saliva from 11 children diagnosed positive by NSP (nasopharyngeal swab). They used the Allplex 2019-nCoV Assay kit for the detection of SARS-CoV-2. Eight children tested positive after saliva testing, and in most patients, the amount of RNA in the saliva decreased quickly over time. Thus, the positivity in saliva decreased from 80% in the first week to 33% in the second week and to 11% in the third week [24]. Another study carried out on a group of 11 children, of which six were asymptomatic and five were symptomatic, reported the detection of the SARS-CoV-2 virus in at least one specimen from the saliva of nine out of 11 children, revealing a trend that the specimens from the saliva should contain less viral loads than those from the NP. Starting with the 8th day after the diagnosis, SARS-CoV-2 was no longer identified in the saliva specimens, but it continued to be detected in the NP specimens. The authors concluded that SARS-CoV-2 can also be detected in the saliva samples of children with COVID-19, but due to the fact that they have a lower sensitivity than NP samples, they are not recommended for screening the disease in children [25].

### 3.2. Alterations of the Oral Mucosa

The oral mucosa is permanently exposed to external factors and represents a true barrier of the body. It also represents a place where general inflammatory diseases as well as autoimmune diseases frequently manifest [26]. Fibroma, gingivitis, stomatitis, aphthous ulceration, cheilitis, and ulcer are some of the most common lesions that can affect the oral mucosa [27]. In adult patients diagnosed with COVID-19, a series of lesions on the oral mucosa were identified, but it was not precisely determined whether they appeared as complications of the disease [28]. Among the most common oral lesions discovered in patients with COVID-19 are erythematous plaques, ulcers, blisters, bullae, petechiae, mucositis, and desquamative gingivitis, which are localized on the tongue, palate, gums, or buccal mucosa [29]. Although it is not known exactly if these lesions appeared as a manifestation of COVID-19, many oral lesions are manifestations of viral diseases, so they can also be caused by COVID-19 and should be known [28].

Several articles have been identified in the literature that reported the presence of oral lesions in children and adolescents diagnosed with COVID-19. Neskovic et al. (2021) presented the case of a 2-year-old female patient diagnosed with COVID-19. The general symptoms were mild, but the patient developed an inflammatory gingivitis located at the level of the maxillary right incisors and at the level of the maxillary canine, but she did not present pain or bleeding. After a week, white discoloration appeared on the upper and lower lip mucosa but also inflammation on the inner lower lip and upper lip frenum. The oral manifestations disappeared within a few days, and a diluted solution of panthenol was applied as treatment 3 times a day [30].

The case of a 9-year-old patient from Iran was also identified. The patient had blisters and erosions located on the tongue, lips, and buccal mucosa accompanying the general symptoms since the onset of the disease. The authors pointed out that oral manifestations could be a sign of a SARS-CoV-2 infection, especially in children [31].

Sometimes, oral manifestations precede the onset of fever, malaise or gastrointestinal symptoms. A case report presented the situation of a 9-year-old boy, who had oral ulcers and swollen lips, and after 24 h, the general symptoms of COVID-19 started. The oral symptoms improved in a few days, but the general condition worsened, and the patient was finally admitted to the pediatric intensive care unit [32].

Bowe et al. (2021) described the cases of two male patients diagnosed with COVID-19 who had conjunctivitis and manifestations in the oral cavity. The first patient, aged 17, developed ulcers in the oral cavity 6 days after the onset of general symptoms, and the second patient, aged 14, developed ulcers in the oral mucosa 4 days after the onset of general symptoms. Both patients denied the use of any medication before the occurrence of oral lesions [33].

A retrospective study that aimed to investigate the prevalence and characteristics of oral and skin lesions in a group of Italian children diagnosed with COVID-19 was identified. The sample consisted of 27 children aged between 3 months and 14 years. Overall, 2 patients presented pseudomembranous candidiasis, 1 patient presented geographic tongue, 2 patients presented coated tongue, and 10 patients presented hyperemic pharynx [34]. At the level of the oral cavity, pseudomembranous candidiasis presents itself as an acute infection, appearing as whitish confluent plaques, producing mild symptoms and appearing frequently in extreme ages, immunosuppressed patients, diabetics, and patients who have used broad-spectrum antibiotics, but it can also accompany other general pathologies [35]. Geographic tongue is a lesion of unknown origin that can accompany various intraoral and extraoral diseases [36], and coated tongue manifests itself as a grayish-white deposit on the tongue, and it is the main cause of the occurrence of intraoral halitosis [37].

Although the COVID-19 literature regarding neonates is scarce, an article that presented three cases of neonates diagnosed with COVID-19 in the first 2 weeks of life was published. All patients were full term and received an APGAR score of 10 at 1 min. The first patient, male, tested positive but developed mild general symptoms. The second patient, male, and the third, female, developed a more pronounced general symptomatology. The oral cavity manifestation, in all three patients, was oral candidiasis, which was treated with Nystatin. All three patients were discharged in the end and did not present any more signs of the disease [38].

To sum up, in children and adolescents without any underlying condition, COVID-19 could determine oral manifestations, such as inflammatory gingivitis [30], blisters, erosions [31], ulcers [32,33], swollen lips [32], candidiasis [34,38], geographic tongue, and coated tongue [34].

### 3.3. Oral Manifestations in COVID-19 Related Multisystem Inflammatory Syndrome and Kawasaki Disease

It has been observed that in pediatric patients, COVID-19 is associated with various general manifestations, with an increasing number of cases in which pediatric patients with mild or asymptomatic forms of COVID-19 have developed a severe systemic inflammatory response, with fever, with the implication of one or several organs, and with a higher risk of death [39]. This condition was initially named pediatric inflammatory multisystem syndrome temporally associated with severe acute respiratory syndrome coronavirus 2 (PIMS-TS) [40], and later, with the increase in the number of cases of children infected with SARS-CoV-2 who presented multisystemic inflammation, the name was changed to multisystem inflammatory syndrome in children (MIS-C) defined by the Center for Disease Control and Prevention (CDC) as the pathology that occurs in a patient under the age of 21, infected with SARS-CoV-2, and who develops fever, inflammation detectable through lab testing, with the involvement of at least two systems, and the general condition of the patient suggests a severe pathology that makes hospitalization mandatory [41]. The involvement of other systems is frequently associated with abdominal pain, conjunctivitis, vomiting, respiratory symptoms, diarrhea, myocarditis, or neurological symptoms [42]. The incidence of MIS-C remains low, with various studies reporting an incidence of 2 per 100,000 inhabitants under the age of 21 [43], 11.4 per 100,000 inhabitants under the age of 20 [44], or 5.1 per 1,000,000 in people under the age of 21 [45], but the success of the treatment depends on the rapid diagnosis of this syndrome [39].

Oral manifestations in children with MIS-C have been insufficiently addressed by the scientific community. Sobh et al. (2022) described the case of an 18-month-old female patient who was brought by her parents because she had a fever and an altered general condition. At the level of the oral cavity, the patient had ulcers, and on the face, a maculopapular erythematous rash. The general condition of the patient worsened and she was finally diagnosed with MIS-C. The patient developed pulmonary hemorrhage, bleeding from the orifices, severe pancytopenia and finally died [46].

Al Ameer et al. (2020) presented the case of a 13-year-old female patient who was diagnosed with COVID-19 and remained asymptomatic for 23 days. On day 23, she started to develop fever, abdominal pain, and sore throat as well as other gastrointestinal symptoms, and she was finally admitted to the hospital and diagnosed with MIS-C. In addition to the aforementioned symptoms, the patient also had skin rash, conjunctivitis, and the oral manifestations that were represented by erythematous, cracked lips. The evolution of the disease was negative, and on the 6th day after hospitalization, the patient died due to cardiac arrest [47].

Apart from these case reports, an original article that presented the oral manifestations of COVID-19-related MIS-C following the analysis of 47 patients diagnosed with MIS-C was included. The study was conducted on patients hospitalized at Morgan Stanley Children’s Hospital of New York-Presbyterian in New York, USA, between 15 March and 1 June 2020. Out of 150 patients under the age of 21 who tested positive for SARS-CoV-2, 47 were diagnosed with MIS-C. The patients were aged between 1.3 and 20 years, with an average age of 9 years. The symptoms were represented by conjunctivitis, systemic rash, extremity edema, gastrointestinal and respiratory symptoms. Oral and oropharyngeal manifestations were identified in 55.3% of patients with MIS-C and were represented by red or swollen lips (23 patients) and strawberry tongue (5 patients). To date, this is the most consistent article regarding the oral manifestations of MIS-C [48].

Described for the first time in 1967, Kawasaki disease (KD) is defined as a self-limited childhood systemic vasculitis that has a predilection for the coronary arteries [49,50]. The etiology of KD is not fully elucidated, but several theories have been proposed such as the environmental toxin theory, the autoimmune pathogenesis theory, and the superantigen toxin theory, but manifestations such as fever, cervical adenitis, conjunctivitis, rash or erythematous pharynx that disappear spontaneously, and often without treatment, indicate a possible infectious etiology [51]. The most frequent complication of KD is coronary artery vasculitis, which can lead to coronary ectasia and aneurysm. Other complications are represented by myopericarditis, arrhythmias, pericardial effusion, myocardial infarction, and sudden cardiac death [52]. Even if KD is the main cause of acquired heart disease in the US in children, and despite the complications that can occur, mortality is low, of approximately 0.01–0.2% [52]. With the onset of the COVID-19 pandemic, an increase in the number of cases of Kawasaki-like disease associated with SARS-CoV-2 infection was observed [53,54].

Some oral manifestations of KD have also been described. In KD, erythema, dryness, fissuring, peeling, bleeding lips, strawberry tongue, prominent fungiform papillae can be present in the oral cavity [55], and cases with severe implication of the oral and labial mucosa have also been described [56].

Regarding the oral manifestations of KD in patients diagnosed with COVID-19, some case report articles were included in this review. Akca et al. (2020) described four cases of Kawasaki-like disease, two of which also had oral manifestations. The first patient, a 7-year-old boy, presented, among other general symptoms, an erosive hyperemia of the oral mucosa, and the COVID-19 disease had a negative evolution, the patient dying from severe hypoxia. The second patient, a 10-year-old girl, presented changes in the lips and oral cavity as well as one-sided submandibular lymphadenopathy. The evolution was good, and the patient was discharged after 7 days [57].

Jones et al. (2020) described the case of a 6-month-old patient who tested positive for COVID-19. Starting with the fourth day after the onset of the fever, the patient also presented dry and cracked lips, and on the 5th day of fever, in the oral cavity, prominent tongue papilla appeared in addition to other general symptoms. The evolution was good [58]. Labe et al. (2020) presented the case of a patient diagnosed with COVID-19-associated KD who at the time of hospitalization, after 8 days of fever, presented cheilitis, stomatitis, and glossitis in the oral cavity [59].

A retrospective study was identified belonging to Falah et al. (2020), who discovered 10 cases of KD patients among COVID-19 positive pediatric patients admitted between April 2020 and July 2020 at Mayo Hospital, Lahore, Pakistan. The patients were aged between 4 months and 11 years. All patients presented fever, and nine out of 10 patients presented conjunctival changes and changes in the oral cavity. These were not described by the authors [60].

Dentists must be alert and aware of the symptoms of KD, especially in the context of the COVID-19 pandemic. Even if the oral manifestations are, in general, self-limited, cardiac complications can be fatal, and patients with KD may initially address the dentist before going to intensive care units due to the high frequency of oral symptoms [61].

Even though in COVID-19-related MIS-C and KD the oral manifestations could be linked to the severity of the disease, the identified oral manifestations should alert the dental practitioner. The following oral alterations were identified in MIS-C: ulcers [46], erythematous, cracked lips [47], and swollen, red lips [48], while in KD, the following were described: erosive hyperemia of the oral mucosa [57], dry and cracked lips, with prominent tongue papilla [58], cheilitis, stomatitis, glossitis [59].

### 3.4. Oral Manifestations in COVID-19 Related Thrombocytopenic Purpura and Erythema Multiforme

An article showing the occurrence of thrombocytopenic purpura in a female patient diagnosed with COVID-19 was identified [62]. Immune thrombocytopenia (ITP) is a bleeding disorder that is characterized by isolated thrombocytopenia [63] and the appearance of petechiae, purpura, ecchymoses, hematomas and muco-cutaneous bleeding [64]. It can be a primary condition or it can be caused by other general diseases [65]. ITP can be triggered by human immunodeficiency virus (HIV), cytomegalovirus, Epstein–Barr virus or even by the seasonal flu [66]. In the case described by Marinescu et al. (2022), a female patient aged 8 years presented to the emergency department with petechiae and ecchymoses on the body, but they were hemodynamically stable. The patient tested positive for infection with the SARS-CoV-2 virus. On admission, there were petechiae and ecchymoses on the face and oral mucosa but without bleeding. Laboratory results indicated a severe thrombocytopenia and leukopenia. At the level of the oral cavity, in the first 48 h after admission, a wet purpura increased in size, as well as petechiae, was observed despite the general good condition. After the administration of the specific general treatment, the patient was discharged 12 days after the onset of symptoms with petechiae and ecchymosis in remission and without other complications [62].

Labe et al. (2020) described the case of a 6-year-old male patient who tested positive for SARS-CoV-2 infection, hospitalized for painful cheilitis, as the first manifestation of a series of other general symptoms. In the oral cavity, in addition to severe erosive cheilitis, the patient had diffuse gingival erosions and thick hemorrhagic crusts. The diagnosis was erythema multiforme [59], which is an immune-mediated reaction that affects the skin and mucous membranes [67] and which can be caused by infection with herpes simplex virus (HSV), Mycoplasma pneumoniae, other viruses or different drugs [68]. The patient whose case was presented by Labe et al. (2020) was tested negative for both HSV and Mycoplasma pneumoniae. He was discharged without further complications after 2 weeks of hospitalization [59].

### 3.5. Acute Parotitis

The parotid gland is the most frequently inflamed salivary gland, which is a condition known as parotitis. This occurs at extreme ages, in general, but it can be found in patients of any age [69]. During the COVID-19 pandemic, the question was raised as to whether it can cause acute parotitis [70], and later, the cases of some patients who were diagnosed with COVID-19 and developed acute parotitis were described [71,72,73]. Acute parotitis can also appear as a viral complication in mumps infection but also in infections with influenza, adenoviruses, herpes simplex virus, or parvovirus B-19 [74]. Two articles that presented cases of children who developed acute parotitis in the context of an infection with the SARS-CoV-2 virus were included.

Sasithorn (2022) reported the case of a 4-year-old male patient who, 4 days after the diagnosis of COVID-19, developed a swelling on the right side of the face, 2 × 2 cm in size, extending from the preauricular area to the mandible. The swelling was painful, soft and non-fluctuating. Tests for various viruses came out negative, and after a 3-day treatment with a non-steroidal anti-inflammatory, the facial swelling resolved [75].

The case of a 10-week-old male patient, diagnosed positive with COVID-19, who developed a unilateral swelling on the right side of the face, firm to palpation, but, as in the previously presented case, without purulent secretion from the Stenon duct in the oral cavity, was also described. Although initially the swelling reduced its size, 21 days after the first presentation, it worsened and became painful. Because the risk of a superinfection was suspected, an antibiotic was added, and after 9 days, the swelling disappeared [76].

### 3.6. Oral Harmful Habits during the COVID-19 Pandemic

Mental health was under attack during the COVID-19 pandemic; studies show that during the COVID-19 pandemic, the rate of depression increased in the general population [77], while among health workers, the level of stress, anxiety and depression rate increased as well [78]. These issues can determine and worsen other general diseases, or they can initiate different pathological situations [79]. Oral harmful habits are among these pathological situations, which can have different negative effects, depending on the duration, frequency and intensity with which they are performed [80]. A habit is a repetitive action that is performed automatically, and when it involves the oral cavity a habit can produce undesired effects. Oral harmful habits include thumb sucking, finger biting, tongue thrusting, lip biting, bruxism, and mouth breathing [81]. Their prevalence is, In general, high in children [82], and if they are left untreated, they can become predisposing factors for various malocclusions [83].

A study that followed the evolution of these harmful oral habits in the COVID-19 pandemic in preschool children was described. Even if oral harmful habits are an indirect effect of the COVID-19 pandemic, their oral manifestations allow their inclusion in this review. Kolcakoglu and Yucal (2021) included 405 questionnaires in their cross-sectional study, composed of 5 parts, which followed the evaluation of anxiety symptoms observed by parents in preschool children as well as the evaluation of harmful oral habits before and after lockdown. Interestingly, harmful oral habits such as finger sucking, nail biting and lip biting decreased in frequency during the lockdown [84]. This can be explained by the fact that during the lockdown, thanks to the presence of parents at home, intra-family relations improved [85].

COVID-19 influences multiple areas of the quality of life [86], and the effects on general health can last up to 12 weeks after the acute episode ends [87]. Moreover, patients may experience respiratory symptoms, fatigue, and a decrease in quality of life up to 6 months after the end of the acute phase [88]. COVID-19 is a disease that continues to be studied because new observations and new information frequently appear [89]. Although new treatments are constantly being tested and multiple clinical trials are ongoing [90], the best method for managing the pandemic remains prevention [91]. Social distancing, as well as other social isolation measures, or lockdown measures have been useful in preventing the increase in the number of new infections [92], and anti-COVID-19 vaccination provides a high degree of protection against SARS-CoV-2 infection [93].

## 4. Conclusions

Although they occur more rarely, oral manifestations can accompany COVID-19 disease in children and adolescents, as it was shown by the various case reports and original articles that were screened, and they can be a sign of COVID-19. Although not as sensitive as NPS, saliva can be used to detect SARS-CoV-2, especially in the first few days of infection. Although the association of COVID-19 with MIS is not very frequent, a new syndrome has been identified (MIS-C), and the success of its treatment depends on early diagnosis.

## Data Availability

Not applicable.
